# Balancing Security and Privacy: Web Bot Detection, Privacy Challenges, and Regulatory Compliance under the GDPR and AI Act

**DOI:** 10.12688/openreseurope.19347.1

**Published:** 2025-03-24

**Authors:** Javier Martínez Llamas, Koen Vranckaert, Davy Preuveneers, Wouter Joosen

**Affiliations:** 1 DistriNet, KU Leuven, Celestijnenlaan 200A, 3001 Heverlee, Belgium; 2 CiTiP, KU Leuven, Sint-Michielsstraat 6 box 3443, 3000 Leuven, Belgium

**Keywords:** web bots; attacks; defences; privacy enhancing technologies; compliance

## Abstract

This paper presents a comprehensive analysis of web bot activity, exploring both offensive and defensive perspectives within the context of modern web infrastructure. As bots play a dual role—enabling malicious activities like credential stuffing and scraping while also facilitating benign automation—distinguishing between humans, good bots, and bad bots has become increasingly critical. We examine the technical challenges of detecting web bots amidst large volumes of benign traffic, highlighting the privacy risks involved in monitoring users at scale. Additionally, the study dives into the use of Privacy Enhancing Technologies (PETs) to strike a balance between bot detection and user privacy. These technologies provide innovative approaches to minimising data exposure while maintaining the effectiveness of bot-detection mechanisms. Furthermore, we explore the legal and ethical considerations associated with bot detection, mapping the technical solutions to the regulatory frameworks set forth by the EU General Data Protection Regulation (GDPR) and the Artificial Intelligence Act (AI Act). By analysing these regulatory constraints, we provide insights into how organisations can ensure compliance while maintaining robust bot defence strategies, fostering a responsible approach to cybersecurity in a privacy-conscious world.

## 1 Introduction

Web bots, automated programs designed to perform repetitive tasks at high speed, have become indispensable tools in fields as diverse as e-commerce, social media, and digital advertising. By enabling processes such as web scraping, automated customer support, and content indexing, bots allow businesses to achieve significant scale, efficiency, and personalisation. This has led to automated traffic accounting for approximately half of all internet traffic ^1^. However, alongside the growing utility of web bots, there has been a concurrent rise in the proliferation of malicious bots that compromise security, violate privacy, and threaten the integrity of online ecosystems and users. These malicious bots contribute to activities such as account takeovers, Distributed Denial of Service (DDoS) attacks, or web scraping and scalping. To mitigate these risks, developers have increasingly turned to advanced detection systems designed to distinguish legitimate user activity from bot-generated actions.

Despite significant advancements in bot detection technologies, creators of malicious web bots have developed increasingly sophisticated evasion techniques, leading to an ongoing “arms race” between bot detection systems and bot developers. This dynamic is marked by a cycle in which each new advancement in detection technology is met with counter-innovations in evasion tactics. Initially, detection methods relied on basic heuristics, such as IP address blacklisting and rate-limiting. However, bots have since evolved to mimic human-like behaviour, mask their origins using proxy servers, and even employ Artificial Intelligence (AI) to circumvent these defences. This continuous escalation has created a demand for more complex detection systems that incorporate behavioural analysis, device fingerprinting, and advanced Machine Learning (ML) algorithms to more effectively identify and mitigate suspicious activities.

Simultaneously, the imperative to stay ahead in this ongoing arms race has spurred an unprecedented surge in data collection. Modern detection systems often rely on vast amounts of user data—spanning browsing patterns, device characteristics, and other behavioural indicators—to effectively identify and analyse bot activity. The acquisition of such detailed data enables security systems to differentiate between authentic users and sophisticated bots. However, this dependence on extensive data collection introduces significant challenges, particularly concerning privacy and user rights. Collecting, storing, and processing such sensitive information can risk violating user privacy if not managed with strict adherence to regulatory standards.

The regulatory landscape has recently evolved in response to these growing privacy concerns. The General Data Protection Regulation (GDPR) ^2^ in the European Union, which came into effect in 2018, set a new benchmark for data protection, emphasising principles of transparency, data minimisation, and user consent. More recently, the AI Act, which was adopted earlier this year, seeks to establish additional guidelines specific to AI and automated systems, including high-risk applications, in order to safeguard users’ rights and ensure ethical standards in AI deployment. Together, these frameworks reflect a heightened regulatory focus on privacy, mandating that any data collection efforts—including those used in bot detection—adhere to principles that prioritise user control and accountability.

As bot detection systems advance in response to evolving threats, the need to balance these innovations with regulatory compliance has become increasingly pressing. This study presents a comprehensive, data-driven taxonomy of web bot detection methods and examines how web bots can circumvent these techniques. Additionally, we explore the practical implications of these methods and their relationship with regulatory compliance, such as the GDPR and the AI Act. Finally, we investigate the use of Privacy Enhancing Technologies (PETs) to strengthen user privacy. Specifically, we explore how detection methods can be optimised to prevent bot-driven threats while minimising the need for extensive data collection, ensuring alignment with privacy regulations and maintaining robust security.

## 2 Web bot detection methods: a data-driven taxonomy

Web bot detection systems can be classified into three primary categories: rule-based heuristics, statistical methods, and ML-based systems. Although these techniques vary in their methodological approaches for identifying bot activity, they fundamentally rely on similar data inputs. A comprehensive understanding of the impact of these detection systems on individual privacy, societal norms, and regulatory frameworks necessitates an alternative categorisation focused on data sources. This section presents an in-depth taxonomy of web bot detection methods, classifying them according to their primary data source. By adopting this structured, data-centric perspective, we aim to provide a foundational overview of bot detection techniques, outlining each method’s distinct advantages, limitations, and relevance in achieving a balance between security and privacy. This approach is particularly significant in the context of compliance with GDPR and the evolving standards of AI regulation.

### 2.1 Network traffic data

Web bots, analogous to human users, interact with web servers by sending HTTP requests to perform their tasks. Consequently, even in the absence of additional mechanisms, domains inherently possess sufficient data to infer the nature of the interacting entity. Traffic-based analysis emerges as an immediate and versatile approach to web bot detection, offering a continuous stream of information throughout a user’s session. This method allows for real-time monitoring and identification of bot-like behaviour based on patterns in the request and response traffic.

Collected data commonly includes IP addresses and HTTP headers. IP addresses can reveal additional information such as approximate geolocation, Internet Service Provider (ISP) details, or IP type (e.g. residential or data centre), aiding in identifying automated activity. Despite potential inaccuracies, such as misidentification due to shared addresses, IPs remain one of the most prevalent markers in detecting automated bot activity. HTTP headers provide a broader dataset by capturing all interaction traces between the client and the server. While the specific headers collected may vary across websites, certain attributes are widely recognised as indicative markers. These commonly encompass the language setting of the response, the content length of HTTP requests and responses, cookies, content-type, timestamps, and the URL referers. Additionally, headers track details such as request forwarding status and User-Agent information, the latter revealing the type and version of browser or software used.

***2.1.1 Web bot detection methods***

**Rule-based Methods.** IP addresses serve as a foundational metric for identifying automated traffic patterns on websites. Detection systems leverage IP-based heuristics to discern user behaviour, with a common approach involving the tracking of request frequency from individual IP addresses over specified time intervals. A surge in requests from the same IP address within a short period, often exceeding humanly feasible rates, typically indicates automation. Rule-based systems also utilise IP geolocation data to assess the probability of bot behaviour. Automated traffic is frequently routed through data centre IP ranges, proxies or Virtual Private Networks (VPNs) rather than residential networks ^3– 6^, as later discussed in Section 3.2.3. By cross-referencing IP addresses with databases of known data centres, VPNs, and proxy servers, these systems can filter out traffic likely originating from bot networks attempting to circumvent detection through non-residential sources. Additionally, such systems maintain and regularly update blacklists of IP addresses associated with past malicious or automated activity, creating a historical framework for recognising probable bots based on prior patterns of abuse ^7^.

HTTP requests can reveal inconsistencies or unusual patterns indicative of automated behaviour. Rule-based systems typically analyse headers such as the User-Agent, Accept-Language and Referer to detect deviations from expected human norms ^8^. For instance, the User-Agent header, which identifies the device, browser, and operating system of a visitor, is often used as a first line of detection. Bots commonly employ default or generic User-Agent strings that do not match typical human patterns or may attempt to mimic human agents with slight inaccuracies. Detection systems can cross-reference these User-Agent strings against known lists of legitimate or suspicious agents, flagging those that are uncharacteristic of genuine traffic. Similarly, inconsistent or missing language headers may suggest bot traffic, as automated tools sometimes omit these details or use standardised values that do not match expected geographic or user-specific patterns.

Additionally, web servers commonly employ a *robots.txt* file, which specifies a list of paths within the domain that are prohibited for web bots to access. Legitimate bots are expected to retrieve the file and adhere to its guidelines before crawling a site. Consequently, entities that access *robots.txt* are typically labelled as bots, while malicious bots bypass this file to avoid detection and access restricted content.

**Statistical Methods.** By collecting and analysing large volumes of traffic data, statistical models can establish a baseline of normal user behaviour, making it possible to detect deviations that suggest automated activity. Contrary to rule-based approaches, statistical models analyse global patterns instead of isolated statistics. For instance, the work by Doran and Gokhale ^9^ leverages web access logs to train a discrete time Markov chain (DTMC) that extracts the differences in the resource request patterns of robots and humans. Similarly, Wei-Zhou Lu and Shun-Zheng Yu ^10^ propose Hidden Markov Models to distinguish the type of activity based on request arrival patterns. Suchacka and Sobkow ^11^ utilise web traffic from an e-commerce site and a set of aggregated features, such as the number of pages visited or HTTP requests, the volume of data retrieved, the duration of the session, time spent in a page, and whether they performed certain actions (e.g. purchase a product or try to log-in) to model the Bayesian probabilistic distribution of humans and bots.

**Machine Learning-based Methods.** Approaches utilising ML techniques demonstrate substantial variation in the selection of relevant session features, the techniques applied, and the methodologies employed for session extraction and the evaluation of classifiers in experimental settings. Traditional algorithms such as Random Forests, Support Vector Machines (SVM) or *k*-Nearest Neighbours ( *k*-NN) have been a recurrent approach for malicious web bot detection ^12– 15^, with models trained on raw or engineered features derived from HTTP requests and access logs and a focus on accuracy. Lagopoulos and Tsoumakas ^16^ explore this idea but aim to capture the semantics of the content of the requested resources. Alternatively, additional works ^17^ focus on the responsiveness of the systems, prioritising detection haste and minimising the number of requests needed to make a decision. More complex Deep Learning models are also employed for bot detection ^18, 19^, including adaptive defences via Reinforcement Learning (RL) algorithms ^20^ that self-improve against new adversaries and evasion techniques.

### 2.2 Fingerprints

Traffic analysis-based detection presents a significant challenge: it necessitates multiple user interactions to determine whether the activity is automated or human. An alternative approach employs the use of fingerprints, which can detect, in a single initial request, the type of user. These fingerprints are composed of a set of attributes that, when combined, generate a unique identifier for an individual user. While fingerprints have been historically associated with tracking ^21– 25^, they can also serve security purposes by conducting a pre-screening of users to identify potential bot-like traits ^8, 26, 27^. However, despite their potential applications, privacy concerns persist, wherein the same data could be repurposed for individual tracking at any given moment. This dual-use dilemma is a recurring challenge in web bot detection systems, where the collection of more data enhances the system’s ability to distinguish between humans and bots, yet simultaneously increases the risk of transgressing user privacy. This is exemplified by the use of IP addresses, which can form a unique fingerprint. Although IP addresses can be leveraged for one-shot detection, they also facilitate individual tracking over time.

**Browser Fingerprints** leverage the distinctive characteristics of a user’s web browser to generate a persistent—not necessarily *unique*—identifier. Commonly used features in browser fingerprinting include the user agent string (as previously mentioned), as well as the operating system and platform; screen resolution and colour depth; installed fonts; time zone and language preferences; and support for specific web technologies such as WebGL, Canvas, and JavaScript features ^8, 28^. These attributes (see Table 1) are typically collected through JavaScript code running in the user’s browser, which gathers the information during page load and transmits it to the server for analysis. Naturally, the absence of JavaScript support can itself serve as an indicator of bot behaviour.

**Table 1.** Selected Examples of Fingerprint Attributes Collected by a Web Bot Detection System.

Name	Description	Example
User-Agent	Identifies the browser and operating system used by the client.	Mozilla/5.0 (Windows NT 6.1; Win64; x64; rv:47.0) Gecko/20100101 Firefox/47.0
Timestamp	Records the time when the request was made.	2024-12-22 14:00:00
Timezone	Tracks the timezone setting of the client’s system.	UTC+02:00 (Europe/Paris)
IP	Tracks the network location of the client.	192.168.1.1
Plug-ins	Detects the set of plug-ins or extensions active in the browser.	uBlock, Grammarly, LastPass
Canvas	Unique fingerprint produced by the HTML5 canvas element.	Hash value
Screen Resolution	Identifies the screen resolution of the client’s device.	1920x1080
Viewport	Identifies the visible area of the web page in the browser.	1920x883
GPU	Identifies the graphics card used by the client.	NVIDIA GeForce RTX 2060
Fonts	Tracks the fonts installed on the client’s device, providing a unique fingerprint.	Arial, Helvetica
Language	Identifies the language settings in the browser or operating system.	en-GB
Cookie Support	Detects whether cookies are enabled or supported by the client’s browser.	Boolean value
JavaScript Support	Verifies if JavaScript is enabled in the client’s browser.	Boolean value
Touch Screen Support	Detects whether the client’s device has touch screen capabilities.	Boolean value, touch points: 2
WebDriver	Identifies the presence of automated browsing tools like Selenium.	Boolean value

**Device Fingerprints** utilise the properties of a device. Key features commonly employed in device fingerprinting include the device’s operating system, model, time zone, touch support, language, screen resolution, and other hardware-specific attributes such as GPU and CPU details, battery status, and sensor data ^21, 29, 30^. As in the case of browser fingerprints, these are collected through client-side JavaScript.

**TLS Fingerprints** identifies and tracks users or detect bots based on the unique characteristics of their Transport Layer Security (TLS) handshake. During a TLS handshake—established when a user’s device connects securely to a server—a variety of parameters are exchanged, including cipher suites, TLS version, supported extensions, and specific ordering of these elements ^31– 33^. These parameters are distinctively configured for different devices, operating systems, and software versions, allowing servers to identify anomalies or non-human behaviours. Because legitimate browsers and devices tend to have predictable, consistent TLS fingerprints, unusual configurations or changes in the TLS handshake pattern can indicate the use of automation tools, proxies, or bot frameworks, which often lack the fine-tuned settings of typical user devices. Nonetheless, changes in TLS libraries or updates to security protocols can complicate its reliability over time.

***2.2.1 Detection methods***

For device fingerprints, detection primarily relies on rule-based systems; however, these attributes can be used in conjunction with ML models to enhance the quality and diversity of training data. Rule-based systems cross-reference fingerprints with a set of rules calibrated to recognise known patterns of legitimate user behaviour and typical device configurations ^8, 25, 34^. The extent of attribute collection and the efficacy of rule-based detection mechanisms are contingent upon the particular organisation or defence system implementing them.

Rule-based systems offer efficient and reliable accuracy in bot detection. However, like other rule-based frameworks, they face two significant limitations: rigidity and the high maintenance costs associated with rule databases. These systems are tailored to address only *specific* and *known* scenarios, limiting their adaptability to adaptive or novel threats. Consequently, commercial and proprietary web bot detection solutions do not disclose the complete list of collected fingerprints or the specific details of their processing methods. Instead, they generally indicate only that such attributes are collected and subjected to analysis. This approach prevents malicious bots from altering their fingerprints to match those of a benign user, as will be discussed in Section 3.

### 2.3 Behavioural biometrics

Network traffic and digital fingerprints constitute the most extensively utilised data types for web bot detection, favoured for their accessibility, ease of collection and analysis. However, it is essential to recognise that users typically interact with websites not through direct HTTP requests but rather through a manual, visual navigation of a webpage. This interaction comprises a series of nuanced behaviours, including mouse movements, scrolling, clicks, and keystrokes. Capturing these behavioural patterns introduces an additional layer of complexity, as they are challenging for web bots to replicate authentically due to the bots’ inherently coarse or repetitive nature.

Mouse movement data captures how users explore and interact visually with a webpage. Human users typically exhibit non-linear, erratic cursor paths, with pauses and shifts that reflect attention and engagement. In contrast, bots tend to produce linear, highly efficient cursor paths with movements at fixed intervals, betraying their automated nature. Similarly, scrolling behaviours differ: human users adjust scrolling speed and position dynamically based on content interest, while bots usually scroll at steady rates or set increments, signalling automation. Additionally, keystroke dynamics offer further detection insights by revealing individual typing patterns. Humans show variability in speed, rhythm, and occasional errors, whereas bots tend to produce keystrokes with uniform timing, lacking the natural inconsistencies characteristic of human interaction.

However, this approach raises significant concerns. Behavioural biometrics, by its nature, can reveal unique identifiers tied to individual users. The detailed nature of this data may inadvertently expose information that could be exploited for tracking or profiling purposes. For example, subtle, user-specific traits in keystroke dynamics or mouse movements can function as a *de facto* digital fingerprint, facilitating identification across different sessions or websites. This introduces privacy risks, as the accumulation of such data could be used to infer sensitive aspects of a user’s behaviour, preferences, or even identity.

***2.3.1 Detection methods***

Historically, detection systems relied on human cognitive superiority over bots, assuming it would be challenging, if not virtually impossible, for bots to replicate human capabilities. This rationale was the inspiration for CAPTCHAs or “Completely Automated Public Turing test to tell Computers and Humans Apart” ^35^, which presented users with challenges—such as text recognition—that only humans were expected to solve. However, the recent advancements in AI have dismantled this paradigm, as models now perform tasks at which humans were inherently superior, including visual recognition. Consequently, detection methods are increasingly incorporating machine learning models to analyse and model user behavioural biometrics.

Mouse dynamics have long been a focus in biometric research ^36– 38^. Their application in bot detection, however, is a more recent development ^39– 43^. Wei *et al.* ^43^ present an approach that transforms mouse movements into images encoding spatial and kinematic information, enabling the use of Convolutional Neural Networks (CNNs), which are extensively applied in image recognition. Iliou *et al.* ^41^ extends this idea by integrating two distinct models—one trained on web access logs and another on mouse movement data with CNNs—creating a comprehensive system for web bot detection. Similarly, Niu *et al.* ^42^ explores different techniques to encode mouse dynamics as images. Alternatively, other studies ^40^ treat mouse dynamics as temporal data, leveraging Gradient Boosting Decision Trees and Long Short-Term Memory (LSTM) networks to capture sequential patterns for bot detection.

Although keystrokes, as with fingerprints or mouse movement, have been traditionally used for authorisation and identification ^44– 46^, recent studies explore keystroke dynamics as an alternative to mouse dynamics for bot detection ^47, 48^. In these approaches, researchers train various supervised models, including SVM, Naïve Bayes, and LSTM networks, to identify bot activity using synthetic data. Keystrokes are modelled as time-series data, allowing for the capture of temporal patterns characteristic of human versus automated input.

## 3 Advanced web bot techniques and evasion strategies

As previously discussed, the landscape of web bots has evolved into a continuous arms race. Understanding the trends and priorities in web bot detection systems necessitates an analysis of both the intent and capabilities of web bots. By exposing the vulnerabilities and limitations of detection techniques, the increasing reliance on more aggressive data collection practices, along with the accompanying privacy concerns, becomes apparent. This section provides an overview of the current bot landscape, emphasising state-of-the-art and state-of-practice evasion techniques.

### 3.1 An overview of web bots

Automated traffic can be categorised into two primary groups based on its intent: good bots and bad bots. Good or benign bots are designed to improve user experience, streamline web interactions, and automate repetitive tasks, such as search engine indexing and content aggregation. These bots enhance the efficiency and accessibility of the internet by enabling faster data retrieval and improving online services. They typically do disclose their automated nature and are expected to comply with the directives outlined in the *robots.txt* file. For example, Google’s web crawler, which is used for indexing pages for its search engine, identifies itself through the user agent “Googlebot”.

In contrast, malicious bots, often referred to as bad bots, are deployed for harmful purposes. These bots engage in activities such as data scraping, scalping, spamming, credential stuffing, or executing distributed denial-of-service (DDoS) attacks. They compromise website security, degrade user experience, and can result in significant financial and reputational harm. Consequently, while good bots are generally accepted—for instance, some webpages may prefer to restrict data scraping for training large language models (LLMs)—malicious bots are subject to targeted countermeasures. While good bots account for approximately 20% of all internet traffic ^1^, the total share of malicious bots corresponds to roughly 30%, with the remaining 50% being attributable to human traffic.

Bad bots can, in turn, be categorised into three groups based on their level of sophistication: simple, moderate, and advanced. Simple bots are characterised by their lack of deception capabilities and do not disclose their automated nature. These may include simple Python scripts or bash-automated cURL commands. In contrast, moderate bots attempt to disguise themselves as legitimate browsers by altering their user agent and incorporating browser-like functionalities. These include headless browsers with JavaScript capabilities. Advanced bots further enhance evasive techniques by emulating human behaviours, such as mouse movements, clicks, or human-like browsing patterns.

### 3.2 Adversarial and evasion techniques

This subsection explores the key strategies used by advanced web bots to evade detection, highlighting the challenges faced by web administrators and security professionals in their efforts to protect websites from malicious automation.

***3.2.1 Headless browser***

A headless browser, in contrast to a headful browser, is a web browser that operates without a graphical user interface (GUI), meaning it does not display visual elements such as buttons, images, or text on the screen. Instead, it runs in the background, executing the same web interactions as a standard browser but without rendering content for user viewing. Headless browsers are commonly used for automated tasks such as web scraping, testing, and performance monitoring, as they consume fewer resources and can execute tasks faster. Automation frameworks like Selenium ^I^, Puppeteer ^II^, or Playwright ^III^ are popular implementations of headless browsers, offering powerful scripting capabilities to interact with web pages. While headless browsers serve legitimate purposes, they are also exploited by malicious actors to bypass detection mechanisms. As a result, they have become the *de facto* tools for bot development. The programmatic and granular nature of automation frameworks and headless browsers allows for the simulation of complex user interactions, support for JavaScript, and the crafting of adversarial fingerprints. Subsequent evasion techniques are built upon these frameworks.

***3.2.2 Adversarial fingerprints***

To prevent abuse, headless browsers typically disclose their automated nature by default ^28^. For example, headless Google Chrome includes the substring "HeadlessChrome" as part of the user agent. However, this disclosure can be easily bypassed, as the same tools that enable headless browsing also allow for the customisation of user agents. Similarly, Chromium’s rendering engine can reveal that the browser is being automated, circumvented by disabling a flag, — disable-blink-features=AutomationControlled.

By following these principles, a malicious actor can alter a headless browser’s configuration to the extent that its fingerprints become indistinguishable from those of a human-controlled one. In response, security measures continuously update the list of the attributes and their values to detect and counter such evasive tactics. A more comprehensive list of Chromium’s Command Line Switches can be found online ^IV^, as well as through Firefox’s configuration ^V^.

***3.2.3 Network proxies and IP rotation***

As previously discussed, IP addresses are a primary defence mechanism against web bots. By comparing incoming IP addresses against databases of blacklisted IPs associated with prior malicious activity, websites can directly block access from these sources. In response, automated traffic began to leverage network proxies. A network proxy serves as an intermediary between a client and a server, forwarding requests while concealing the client’s true IP address. By routing traffic through multiple proxies, bots can anonymise their activities and simulate requests from diverse geographical locations ^4^. These proxies are typically sold by companies and include data centre proxies and residential IP proxies (RESIPs). Data centre proxies provide IP addresses that are not affiliated with ISPs and originate from third-party cloud service providers. While they are cheaper, they are easier to detect and are typically used for less sensitive tasks or those with a low risk of blocking. In contrast, RESIPs route traffic through networks of genuine residential devices and real IP addresses assigned by ISPs to residential homes; helping to bypass IP-based bans and other anti-bot measures ^3, 5^. However, the use of RESIPs can raise ethical concerns if the IPs are obtained without the consent of end users, as some services may route traffic through residential devices without full transparency ^49– 51^.

Building on this concept, IP rotation dynamically changes the IP addresses used for each request or batch of requests, often by utilising pools of proxy servers. This approach is particularly effective in bypassing rate-limiting mechanisms and evading blocks that rely on identifying excessive requests originating from a single IP address.

***3.2.4 Rate limiting and throttling***

Advanced bots employ sophisticated techniques to bypass rate limiting and throttling mechanisms, which are designed to regulate the volume and frequency of requests sent to a web server. Rate limiting, a widely used defence mechanism, detects and blocks excessive requests, often indicative of bot activity. Throttling, a closely related concept, involves deliberately slowing down incoming requests to prevent servers from being overwhelmed.

To circumvent these measures, bots can distribute their requests across multiple IP addresses using proxy networks or IP rotation. By ensuring that no single IP address exceeds the permitted request limit, bots effectively evade rate-based restrictions. Additionally, bots may implement request scheduling algorithms to vary the timing and frequency of their interactions. By introducing random delays between actions, bots mimic human browsing patterns, thereby reducing the likelihood of triggering detection systems that analyse request patterns over time.

Similarly, bots can repeatedly generate new user sessions, each associated with a unique set of cookies, headers, and user-agent strings. This makes it challenging for websites to identify and correlate requests originating from the same bot. Additionally, advanced bots can monitor server responses in real time, dynamically adjusting their behaviour to avoid detection thresholds. For example, if a server issues warnings or temporary blocks, the bot can reduce its request rate or temporarily halt activity before resuming operations under different parameters.

***3.2.5 Behavioural mimicry***

This strategy involves programming bots to replicate user-like behaviours, such as typing speeds, mouse movements, scrolling, and click patterns, to bypass detection systems that analyse behavioural anomalies. By mirroring genuine interactions, bots can evade traditional security measures that rely on identifying non-human activity.

For example, bots may simulate random delays between clicks, navigate websites in a non-linear fashion, or interact with dynamic elements such as drop-down menus and sliders to mimic exploratory behaviour typical of human users. Some bots even simulate real-time decision-making by introducing variability in their interactions, such as hesitating before clicking a button or revisiting a previous page. Advanced implementations use libraries or Machine Learning models ^52^ that generate synthetic mouse and keyboard events to closely replicate human input.

Behavioural mimicry can also extend to session-level actions such as maintaining active sessions over extended periods, performing periodic interactions to prevent timeouts, and even logging out and back into accounts to simulate genuine user workflows. Furthermore, bots can take contextual cues into account, such as changing their behaviour based on the type of content displayed on a page or responding dynamically to errors or timeouts. These strategies significantly challenge detection systems, as the bots are designed to blend seamlessly with legitimate traffic, reducing the effectiveness of anomaly-based detection methods.

***3.2.6 Machine Learning***

Leveraging Machine Learning, advanced bots analyse patterns in detection systems, learning or adapting their strategies in real-time to maintain effectiveness. This capacity to learn from previous interactions renders adaptive bots highly resilient and capable of evading even sophisticated defence mechanisms.

For instance, adaptive bots can monitor server responses to detect potential indicators of blocking, such as increased latency or warning messages. Based on these observations, the bot can dynamically adjust its behaviour by reducing request rates, switching IP addresses, or altering request headers. Some bots are further equipped with reinforcement learning algorithms ^53^, enabling them to test multiple strategies and prioritise those that yield the highest success rates, thereby optimising their behaviour over time or respond to new defences. Similarly, AI-powered techniques enhance the ability of web bots to bypass CAPTCHA challenges ^54^.

## 4 Privacy challenges and regulatory compliance with GDPR and AI Act

Web bot detection on online platforms comes with several challenges that may affect user privacy. First, bot detection comes with the monitoring of several sources of information regarding the user, many of which are personal data and/or allow inference of personal data regarding said user. Bot detection thus has the potential to be a means for constant surveillance. Second, an automated bot detection system can affect the rights of users if it feeds into automated decision-making mechanisms, such as automated responses. Both false negatives (bots being allowed to pass through) and false positives (legitimate users are falsely accused of being bots and attackers) can result in decisions that affect users’ lives in ways that infringe on their fundamental rights. For example, false positives can result in measures such as charging an individual with attacking, suspending online accounts (and thus suppressing legitimate speech or legitimate system use) or reporting legitimate behaviour as illegitimate to authorities.

The EU legislator has been active in the past years in updating regulations and providing additional regulations to ensure that users’ privacy and other fundamental rights are respected when they make use of digital technologies. To ensure user privacy, especially the General Data Protection Regulation 2016/679 (hereinafter “GDPR”) ^VI^ and the Artificial Intelligence Act 2024/1689 (hereinafter “AI Act”) ^VII^ are relevant. In this chapter, we discuss the application of the aforementioned regulations to web bot detection and the automated responses that may come from it.

### 4.1 Web bot detection under the GDPR

***4.1.1 Personal data in the context of web bot detection***

Personal data (article 4(1) GDPR) is any information relating to an identifiable individual, the “data subject” and can contain identifiers related to that person or information about that person. According to Recital 26 GDPR, whether or not an individual is identifiable depends on all the means the controller or another person can reasonably use to identify them, including by singling them out in a group. This includes the costs and the amount of time required for identification, as well as available technology. Note that the identifiability of an individual based on personal data depends on the relative means that are available to the controller.

In *Nowak* ^VIII^, the Court of Justice of the European Union (CJEU) confirmed that the concept of “personal data” must be given a wide scope that encompasses all information, whether or not it is sensitive or private, objective or subjective, as long as it is connected to a natural person by reason of its consent, purpose or effect. In *Breyer* ^IX^, the CJEU confirmed that IP addresses, including dynamic IP addresses, can be personal data, unless if identifying a natural person based on their IP address would be prohibited by law or practically impossible on account of requiring “a disproportionate effort” in terms of time, cost and manpower, resulting in an insignificant risks of identification. By “effort”, the CJEU refers to the effort on the part of the controller. The interpretation in *Breyer* has been confirmed in more recent case law and extended to other categories of data, such as vehicle identification numbers (VINS) ^X^ ^55^. This relative concept means that, to most ISPs, IP addresses (and likely other device identifiers) constitute personal data, since they have the technological capacity to single out individuals based on them ^55^. As AI analysis tools are set to increase the capacity to identify individuals based on seemingly anonymous data, what constitutes “personal data” is set to further expand.

In *OT* ^XI^, the CJEU equally confirmed that data that can be inferred, equally constitutes personal data. With the increased proliferation of data analytics and machine learning, the types of data that can be related to one’s device and thus to a natural person themselves, is set to increase further, as more entities have more advanced means of analysis at their disposal.

Applied to the web bot detection methods outlined above, one must conclude that all data that is being monitored is potentially personal data of a user, which, in combination with other data that may be available or with easily available computing resources, constitutes personal data. For all personal data processing, the controller—the entity who decides to deploy web bot detection on their systems—will have to ensure that the processing has an appropriate legal basis (Article 6.1 GDPR) and that in all events, the principles of data protection are complied with (Article 5 GDPR).

In terms of legal basis, there are several options. Consent (Article 6.1(a) GDPR) is often not preferred, as it requires a free, specific, informed and unambiguous affirmative action (in other words, ticking a box), as well as that the consent must be as easily revoked as it is given. For security purposes, this is often unworkable. After all, consent can only be given if it is free, which is absent if there is a power imbalance between the data subject and the platform. With many cases of web bot detection, the idea is that the mechanism triggers independently from any consent given by the data subject. In some cases, it might be, however, necessary; especially when related to data that is sensitive under Article 9 GDPR, including biometric data processing (see below).

Consent is also necessary if any bot detection technique makes use of data that any storing of information on the equipment of a subscriber (like a cookie or any other file) is only permitted with the opt-in consent, as required by Article 5(3) ePrivacy Directive ^XII^.

Contractual necessity (Article 6.1(b) GDPR) only grants permission to grant the data that is necessary to either enter into or perform a contract. Examples of such data include shipping details and payment options. For a contract explicitly related to the running of the website, security often goes beyond the mere provision of a service.

Controllers may nonetheless be required to process some data, if it is accepted that web bot detection is a processing method that is necessary to comply with a legal obligation. This appears far-fetched, as there is no general obligation to utilise web bot detection. However, there are more and more legal obligations to ensure that website deployers take “appropriate and proportionate measures” to mitigate the risks of cyber-attacks. The GDPR, for example, requires this of controllers under Article 32 GDPR, as well as the obligation to ensure privacy by design and by default (Article 25 GDPR). The NIS 2 Directive also provides for general risk management measures, which include vulnerability handling and disclosure. The AI Act and Cyber Resilience Act will also require software developers to ensure that security features are built in. Insofar as web bot detection can be based on a best practice or harmonised standard to be the best method to comply with these laws, the legal basis of compliance with a legal obligation may be accepted. Aside from general laws regarding data protection, some sectoral laws can provide more specific obligations. For example, the Digital Operational Resilience Act 2022/2554 requires entities in the financial sector to have mechanisms in place to “promptly detect anomalous activities”, which includes ICT network performance and ICT-related incidents, and may include web bot detection activities as well. ^XIII^

The “catch-all” legal basis for most processing of personal data for the purpose of cybersecurity (including web bot detection), appears to be necessity for the legitimate interest of the controller or third parties (Article 6.1(f) GDPR), provided that the processing is a) necessary for this interest and b) there is no overriding interest or fundamental right of the data subject which requires the protection of personal data ^56^. Recital 47 GDPR lists as examples of some such legitimate interests preventing fraud or direct marketing. Recital 49 GDPR also acknowledges that the processing of personal data “to the extent strictly necessary and proportionate” to ensure network and information security constitutes a legitimate interest of the data controller. Interestingly, Recital 121 NIS 2 Directive provides more elaboration on the matter of the appropriate legal basis of data processing under the GDPR, confirming that processing is usually lawful under Article 6(1)(f) GDPR, or a legal oblication or necessary to carry out a task in the public interest.

Whether or processing is considered necessary for the protection of a legitimate interest, depends on a balancing test that the controller must undertake. This test consists of four steps: a) the controller assesses its own legitimate interest, b) the controller assesses the impact on data subjects, c) the controller measures a provisional balance and d) the controller takes mitigating measures to offset any excessive impact. A key element of such balancing exercise is whether or not the “reasonable expectations” of the data subject are met. For example, if an advanced method of web bot detection is necessary to ensure that systems are not attacked, then the use of web bot detection is justified. If, however, the web bot detection system can be used and is used to profile users in ways that could affect their rights in unrelated contexts, that would clearly violate the requirement of necessity to meet this legal basis.

It must be noted that, for some categories of personal data, the legal basis is set by specific rules. Article 9 GDPR prohibits processing of the following categories of personal data: a) data that reveals the user’s racial or ethnic origin, political opinions, religious or philosophical beliefs, or trade union membership, b) the processing of genetic data ^XIV^, biometric data for the purpose of uniquely identifying a person, data concerning the user’s health or data concerning a person’s sex life or sexual orientation. As an exception, Article 9(2) GDPR provides an exhaustive list of exceptions under which such processing is permitted. The “catch-all” basis is if the data subject has given explicit consent to the processing of such data for the purpose at hand.

This raises questions for the tracking of, for example, mouse movements for the purpose of identifying whether or not a person is a bot in order to ensure the security of traffic on their websites. Biometric data are defined in Article 4(14) GDPR as “ *personal data resulting from specific technical processing relating to the physical, physiological or behavioural characteristics of a natural person, which allow or confirm the unique identification of that natural person, such as facial images or dactyloscopic data*” and consist of, for example, facial patterns, but also gait, mouse patterns, keyboard strokes and the like ^XV^. Mousing patterns or keyboard stroke patterns are thus biometric data. Biometric data are prohibited under Article 9(1) GDPR and thus only allowed under few exceptions, insofar as the processing is done for “the sole purpose of identifying a unique individual”. Identification (i.e. singling out an individual) must be distinguished from biometric authentication and verification. Moreover, it is clarified that if biometrics are used for the purpose of distinguishing one category of people from another, but not identify any single one of them, the processing of said biometric data does not fall under Article 9 ^XVI^. This is thus permissible, provided an appropriate legal basis (usually legitimate interest or legal necessity) is found.

Applied to the above-mentioned web bot detection techniques, this means that the use of mousing patterns, keyboard stroke patterns, etc. to detect bots is in principle allowed for the purposes of network security, provided the necessity can be proven. Consent is, in principle, not required, provided there is no identification of any particular user. All of the above changes if a) mousing patterns are used for identification and/or b) from the data (for example, a list of URLs in combination with mousing patterns and click patterns), other sensitive data can be inferred. With modern techniques, the latter challenge can be easily run into. For example, in the *OT* case, the CJEU held that an organisation was processing sensitive personal data ( *in casu* the data subject’s sexual orientation) by requesting the name of family members on a form. ^XVII^ Other behavioural correlations provide a large potential of data that can be inferred from online behaviour; for example, social media providers advertise based on the likes that a user provides, for example. For the purpose of web bot detection for the mere provision of security, such processing must be excluded.

Regardless of which legal basis is chosen, the principles of data protection under Article 5 need to be complied with. This means that any such processing must be:

- lawful, fair and transparent;
- collected only for a specific purpose (for web bot detection, that would be security of the system);
- adequate, relevant and limited to what is necessary for said purpose;
- accurate and kept up to date;
- kept in a form which permits identification of data subjects no longer than necessary;
- processed in a secure manner.

Once a bot is detected, several decisions can or must be made according to the process. Article 22 GDPR also provides limitations on the use of automated responses. Article 22 GDPR provides a right of the data subject to not be subject to solely automated decision-making, including profiling, which produces legal effects or similarly significant effects concerning him/her.

A “decision” entails that the web bot detection software goes beyond merely supporting a human operator and replaces the human decision-making process. The Article 29 Working Party (predecessor to the European Data Protection Board) does clarify that the human intervention must be “meaningful”: a human merely ticking the box or approving the suggestion without meaningful forms of overruling, does not evade the scope of Article 22 GDPR. ^XVIII^ For example, in *SCHUFA*, the CJEU confirmed that an automated “credit score”, based on an automated solvability scoring system is a form of automated decision-making, if a third party “draws strongly” on that value in their decision to establish a contractual relationship with that person. ^XIX^

However, an automated decision is only subject to the right mentioned above if it meets a threshold of significance. Examples of such decisions include the cancellation of a contract, denial of a social benefit, refused admission to a country or denial of citizenship, decisions that affect one’s financial circumstances, affects someone’s access to health services, employment, education, etc. Recital 71 GDPR does clarify that automated decision-making should be allowed if other EU or national laws permit it, including for fraud and tax-evasion monitoring and prevention, as well as to ensure the security and reliability of a service that is provided by the controller. In any case, there must be adequate human oversight measures in place and automated decision-making may not concern a child. Moreover, any privacy notice also needs to provide “meaningful logic” about any automated decision-making undertaken, in a format and manner that is understandable by the average user (Articles 13 and 14 GDPR).

The restriction on automated decision-making is not applicable if the decision is a) necessary to enter into a contract, b) authorised by law or c) based on the data subject’s explicit consent. In any case, there must be a right to human intervention and to contest the decision. Such decisions may also not be taken on the basis of sensitive data, unless if based on the data subject’s prior consent.

The above grants a permission to controllers to use automated decision-making by web bot detection tools insofar as the use of such automated decision-making is used strictly to ensure the security of the network. This could be used to justify automated responses to bots, which allows defence against cyber-attacks. However, it appears that the GDPR would still require guardrails, including human overruling capacity, in order to ensure that any “mistakes” by the algorithm are still corrected. After all, an automated decision can still result in quite intrusive decisions, especially in the event of a false positive. Think, for example, of a user being blocked from accessing their bank account or a social media business account, because they are unjustly being considered a bot when they are travelling because of the frequency of requests or the location the request comes from. The GDPR, however, by itself, does not give much more guidance on the matter. In any event, there should be a means of human intervention, which can also take place *ex post*.

Some processing operations must be preceded by a Data Protection Impact Assessment (DPIA) undertaken by the controller (Article 35 GDPR). This is a “reflection moment” where the controller documents any risks to the rights and freedoms of individuals from the processing, as well as any mitigating measures that have been taken. DPIAs are necessary when a type of processing is “likely to result in a high risk”. This is in particular the case in three cases: a) a systematic and extensive profiling of natural persons based on automated processing, that produces decisions with legal effects or similarly significant effects, b) processing “on a large scale” of sensitive data such as referred to under Articles 9 and 10 GDPR, as well as a systematic monitoring of a publicly accessible area (Article 35(3) GDPR). In other words: a DPIA must be undertaken if a technology may result in evaluation or scoring that falls afoul of Article 22. If there is little significance to the decision, then this is not required. Whenever data sets are matched or combined, a DPIA is also required. This is also the case when the processing relates to vulnerable individuals, such as children. Any innovative use of technology also means that DPIAs are required ^XX^.

Applied to web bot detection, this means that the need for a DPIA can be pre-empted by observing the principles of data protection by default and by design. This can be achieved by limiting the types of data used to trigger the system, or to make use of a system that is not intrusive in its actions (or, in which the actions are overseen by a human). This can relate to rules-based systems or systems that process any device or browser fingerprints only in order to determine whether or not a device is a bot or not, not to identify the user behind the combination of metadata. Of course, it can still be recommended to conduct a DPIA of one’s own volition.

### 4.2 AI-powered web bot detection and human oversight under the Artificial Intelligence Act

On 2 August 2024, the Artificial Intelligence Act entered into force. Its provisions provide four risk tiers for the design of AI systems in the European Union. These risk tiers consist of a) some AI practices that are banned, b) the requirement to certify some “high-risk” AI systems on certain requirements, as well as certain foundation models c) certain transparency rules and d) voluntary commitments for any AI systems that do not fall within the scope of the above-mentioned systems ^59^.

Article 3(1) AI Act defines “AI systems” as any machine-based system that is designed to operate with varying levels of autonomy and that may show adaptiveness after it is deployed and that can generate outputs from the input it receives, be it predictions, content, recommendations, or decisions, that can influence physical or virtual environments. This definition is a departure from the initial proposal, which provided a closed definition to mostly cover machine learning techniques. While all machine learning is included in the above definition too, the current definition includes all systems that are capable of inferring from inputs. This places other, more rule-based systems, under the scope of application as well.

Nonetheless, not all activities regarding AI systems are subject to the provisions of the AI Act. For example, the AI Act does not apply to any AI systems or AI models that are specifically developed and put into service “for the sole purpose” of scientific research and development (Article 2(6) AI Act). This exemption also covers product-oriented research, testing and development activity (Recital 25 AI Act). It also does not include AI systems that are developed for a mixed purpose (real-world deployment and research). Moreover, the AI Act does not apply to any research, testing or development activity regarding any AI systems prior to their being placed on the market or put into service. Recital 26 does clarify that such research should, in any case, be done in accordance with recognised ethical and professional standards for scientific research and in accordance with “applicable EU law”, such as, for example, the GDPR and respect for fundamental rights.

Article 5 AI Act provides some AI practices which are banned. Most of the bans are of limited relevance to bot detection. For example, Article 5(1)(a) and 5(1)(b) AI Act prohibit any AI system that deploys subliminal or purposefully deceptive techniques, with the objective of effect of materially distorting a person’s behaviour or exploiting their vulnerabilities. When unveiling the initial proposal, the initial examples included an alarm pushing subliminally to work harder than is healthy.

Article 5(1)(c) AI Act does prohibit forms of social scoring, or the evaluation of users based on their social behaviour; insofar as the social score leads to detrimental or unfavourable treatment that is a) unrelated to the context in which the input data was collected or generated or b) unjustified or disproportionate to the behaviour or its gravity. For web bot detection, this can perhaps be met if the online behaviour results in denial of other essential services.

Similarly, risk assessments to determine the risk of criminal activity based on purely automated profiling of a natural person are prohibited. This would only be feasible if the bot detection would result in a charging of an individual with charges based on breaches of anti-hacking legislation.

Article 5(1)(g) AI Act prohibits any biometric categorisation system that categorises individual persons based on their biometric data to infer race, political opinions, trade union memberships, sex life or sexual orientation. If web bot detection is employed in accordance with the GDPR for security purposes only and further function creep is avoided, this prohibition will not be triggered.

The ban on real-time biometric identification systems in publicly accessible places other than by law enforcement authorities (Article 5(1)(h) AI Act) does not provide any limitation on the use of behavioural tracking methods for the purposes of web bot detection. First, the ban applies to biometric identification ^XXI^, but not to biometric verification ^XXII^ or biometric categorisation ^XXIII^ ^60^. Second, Recital 19 AI Act clarifies that “publicly accessible spaces” only refers to physical spaces that are accessible to an undetermined number of natural persons. As online spaces are not covered, they are not physical spaces. Thus, in any case, biometric identification—even in real-time—through the use of AI systems is not forbidden, not by private entities and not by law enforcement authorities. Biometric identification for the purposes of removing bots are thus fully permitted, provided the limitations of the GDPR are permitted.

Some bot detection software with an AI component will, however, be considered a high-risk AI system and thus be required to be certified under the AI Act, albeit for different reasons. Under the AI Act, there are two categories of AI systems that are considered “high-risk”. The first are AI systems that are considered “safety components” ^XXIV^ of products that have already been regulated under the New Legislative Framework, which covers many different hardware products under different sectors. ^XXV^ The second are an exhaustive list of AI systems that are considered high-risk by the EU legislator, by virtue of their inclusion in Annex III AI Act. The first category of such systems are all biometrics, including a) remote biometric identification systems (unless if those systems are AI systems intended to be used for biometric verification, the sole purpose of which is to confirm that a specific natural person is who they claim to be), and AI systems intended to be used for b) biometric categorisation on the basis of sensitive or protected attributes or characteristics or the inference thereof, or c) for emotion recognition.

The second high-risk use case relates to safety components of critical infrastructure under the AI Act. This refers to critical entities as identified under the EU Directive on the Resilience of Critical Entities 2022/2557 (hereinafter referred to as “the RCE Directive”) ^XXVI^. It must be noted that some IT services are now also considered critical infrastructure, including IXP exchange points, DNS service providers, TLD registries, cloud computing services providers, data centre services, content delivery networks, trust services providers and electronic communications services (Annex I RCE Directive). Many online platforms may thus fall within the scope of a high-risk system use case, provided that a Member States designates the platforms using it as a critical entity under the RCE Directive (Article 6 RCE Directive).

Applied to web bot detection, this means that AI-based systems will not require certification, unless if they go into biometric identification. That is, the singling out of individuals, or if they are deployed in critical infrastructures.

Providers of high-risk AI systems have to be certified prior to their placing on the market on their compliance with several requirements. These requirements include the following:

- The provider must have a risk management system in place (Article 9 AI Act);
- Any training, testing and validation data must be subject to “adequate governance and management practices” for the intended purpose of the high-risk AI system. They must also be relevant, sufficiently representative and, to the best extent possible, free of errors and complete in view of the intended purpose, and have “the appropriate statistical properties” in relation to those on whom the AI system is intended to be used. They must also “take into account” the specific geographical, contextual, behavioural or functional setting within which the high-risk AI system is intended to be used. Under some conditions, for the detection of bias, it is legally permitted to process personal data, including sensitive personal data; (Article 10 AI Act);
- The provider must draw up technical documentation and keep it up to date (Article 11 AI Act);
- High-risk AI systems must allow the automating logging of events. For biometric identification systems, this logging must provide, at a minimum, a) the period of use of each system, b) the reference database against which input data was checked, c) the input data which has led to a match and d) the identification of the natural person involved in the verification of results (Article 12 AI Act);
- A high-risk AI system must be designed in such a way that its operation is sufficiently transparent to enable professional users to interpret a system’s output and use it appropriately, which includes a pre-determined set of instructions in an accessible manner which is available in an “appropriate” digital format;
- High-risk AI systems must be designed in a way that allows effective human oversight by natural persons during the time in which they are used. This is to aim any risk to health, safety or fundamental human rights that can emerge during normal use or reasonably foreseeable misuse. This must include either measures built into the design or measures that are implemented by the user. The measures include monitoring of the AI system, including anomaly detection, and can go to overruling the AI system or installing a “stop” button into the system. For biometric identification system, this requirement is to include that at least two persons separately verify before taking a decision, unless in areas where this is considered to be disproportionate (Article 14 AI Act);
- High-risk AI systems must be accurate, robust and secure, as well as resilient against bias in the system and protected against adversarial attacks (Article 15 AI Act).

Moreover, providers are required to have a quality management system in place to ensure compliance with the AI Act’s provisions (Article 17 AI Act).

Compliance is presumed if a high-risk AI system can be certified under a harmonised standard (Article 40 AI Act). If there are no harmonised standards, the EU Commission can adopt “common specifications” to cover the risk category in question (Article 41 AI Act). For bias and cybersecurity, there are also additional certification mechanisms: compliance with one requirement can be proven in a circular way if the data has been selected specifically taking into account the target population. Cybersecurity can be proven if the AI system is certified under the Cybersecurity Act 2019/881 (Article 42 AI Act).

Whether or not compliance with the above requirements can easily be proven, thus depends on the existence and adoption of EU harmonised standards. Whereas the standardisation process is ongoing (CEN and CENELEC are working on it and are scheduled to finalise their work by 30 April 2025), there are concerns that standardisation may not be finished in time, resulting in limited coverage ^61^. This will have impact for the compliance burden for AI Act compliance in Europe.

After all, the presence of harmonised standards determines the conformity assessment procedure. High-risk AI system providers can self-certify for those risk categories for which an EU harmonised standard has been adopted. For those risk categories that there is not—or where the high-risk AI system provider has chosen not to apply it—a third-party conformity assessment body is required to pre-approve placing the AI system on the market, based on an assessment of the technical documentation regarding the model. There is no testing of the model itself. This conformity assessment would be necessary every time there is a “substantial modification” to the AI system in question (Article 25 AI Act). As previously mentioned, not all bot detection systems are high-risk AI systems, as long as they are limited to biometric verification systems and so long as they are not used as safety components of critical infrastructure. Any AI-powered web bot detection system that does meet this scope, however, would be required to be certified, depending on the presence of standards, by a third-party conformity assessment body before they are released on the market. This could make the deployment of such tools more difficult on the EU market. Nonetheless, the fact that the AI model is not tested by itself (only the documentation and the quality management system), some authors criticise the effectiveness of oversight ^62^.

The AI Act’s final version also includes a requirement for the professional user of a high-risk AI system, if they are a deployer of a high-risk AI systems listed in Annex III AI Act and provider of a public service, that user must also conduct a fundamental rights impact assessment of the use of the AI system before putting the AI system into use (Article 27 AI Act). This relates to internal reporting of the description of the processes the AI system will be used in, the period of use, the categories of likely affected persons, the human oversight measures and risk management measures (Article 27 AI Act).

This obligation only applies to the first use of the AI system, but the assessment must be kept up to date. The above could apply to web bot detection, given the risk of impact of false positives on privacy and freedom of expression (see above).

Aside from the rules that apply to high-risk AI systems, some AI systems must comply with “bot disclosure” rules (Article 50 AI Act). For example, the provider must ensure that any AI system that is designed to interact directly with a person must be developed in such a way that the user knows they are interacting with an AI system. Similarly, AI systems that generate synthetic images must have watermarked outputs as well. Users of a biometric categorisation system must also inform the people exposed to that system of the operation of that system, unless if they are permitted by law to detect criminal offences. Similarly, deepfake generators must disclose the artificial nature of the content they produce.

Social bots can nowadays make use of generative AI modelling. When the first Large Language Models were released, the AI Act had to be modified to include specific rules on the use of general-purpose AI (GPAI) models on the EU market. The provider of a GPAI model must provide documentation, information, a policy on copyrights and provide a summary of the content used for the training of the model, unless if these are released under a FOSS license.

Some GPAI models may be considered to pose “systemic risk”. Article 51 defines a model as a GPAI system with systemic risks if a) it has high impact capabilities on the basis of “appropriate” tools and methodologies, or b) if the Commission decides that it has capabilities or an impact equivalent to the benchmarks set out in a). These high impact capabilities are presumed to be present when the training of the model required more than 10 ^25^ FLOPS of computation. Once a model achieves this threshold, the Commission must be notified. Providers of general-purpose AI models with systemic risks are required to perform state-of-the-art model evaluation, assess and mitigate the risks that may stem from the GPAI model, keep track of information about incidents and corrective measures, and ensure an adequate level of cybersecurity. They may rely on Codes of Practice for guidance, such as the one the AI Office released ^XXVII^.

Any other AI systems that do not meet the categories mentioned above, are under no specific safety requirements. Compliance with the requirements for high-risk AI systems is encouraged, not required (Article 95 AI Act). Nonetheless, their use must still respect data protection legislation and any law regarding the protection of human rights.

Web bot detections could be considered AI models with a specific purpose and thus do not fall within the scope of GPAI models. However, if a GPAI model could be used for the purposes of web bot detection, this implies that it may have to meet the above-mentioned requirements, if it is used for the purpose of one of the high-risk AI system use cases mentioned above.

### 4.3 Respecting the intellectual property rights in generated content

As web bot detection using AI also includes the analysis of user-generated content, there is always a risk that there is intellectual property rights on some expressions. Even 11-word sentences by themselves can constitute an original expression, protected by copyright under EU law (CJEU 17 January 2012, C-302/10, Infopaq International A/S v Danske Dagblades Forening.). For the purposes of bot detection, the question has been resolved by the adoption of the Directive on Copyright in the Digital Single Market 2019/790 (hereinafter “CDSM Directive”) ^XXVIII^. Article 4 CDSM Directive provides for an exception for all activities of text and data mining, defined under Article 2(2) CDSM Directive as “ *any automated analytical technique aimed at analysing text and data in digital form in order to generate information which includes but is not limited to patterns, trends and correlations*”. In other words: this also covers the pattern detection that underlies web bot detection activities. This means that, for the purposes of web bot detection, any copyright on the content that is posted is not a major concern, insofar as the behaviour would be copyrighted.

## 5 Technology mapping for privacy-aware web bot detection

Building a web bot detection system that uses sensitive information, such as web server logs (e.g., IP addresses, user agents, URLs) and client-side data (e.g., browser fingerprints), poses substantial challenges in terms of privacy and regulatory compliance. While Privacy Enhancing Technologies (PETs) offer technical solutions to mitigate these challenges, they are insufficient on their own to achieve complete compliance with regulations such as the GDPR and the AI Act. This section provides a comprehensive analysis of how PETs can support compliance efforts, their limitations, and the critical role of organisational measures in ensuring adherence to regulatory requirements.

### 5.1 PETs for compliance

PETs provide technical solutions to protect privacy during data processing. They effectively minimise the risk associated with data breaches, regulatory violations, and re-identification.

***5.1.1 Data Minimisation***

Data minimisation is a fundamental principle of data protection, limiting the collection, processing, and storage of data to what is strictly necessary for its intended purpose. This principle is enshrined in Article 5(1)(c) of the GDPR, which states that personal data must be “adequate, relevant, and limited to what is necessary in relation to the purposes for which they are processed”.

In the context of web bot detection, this entails that the system collects only the strictly necessary data to determine whether a user is a bot. For instance, the system should avoid storing unnecessary identifying information, such as full IP addresses, when an aggregated or hashed form will *suffice*. Notably, this definition is, from a security perspective, far from trivial. As malicious actors take the lead in an ongoing arms race, organisations can never guarantee full protection, and may always justify the need to collect additional data in pursuit of enhanced security. Similarly, as mentioned in previous sections, regulations preclude strict data minimisation, as organisations are mandated to ensure security.

Web bot detection frequently relies on data such as IP addresses to identify suspicious geographic patterns. However, collecting full IP addresses is sensitive, as they are classified as personal data under the GDPR due to their potential to identify individuals when combined with other information. By adhering to the principle of data minimisation, the system can mitigate risks by processing IP addresses in a less sensitive manner, such as removing the last octets or applying hashing techniques to irreversibly transform them. For instance, with octet truncation, an IP address such as 134.58.1.123 is transformed into 134.58.0.0. This approach reduces the risk of identification, as it becomes significantly more challenging to identify an individual using an aggregated address. However, this method has the drawback of reduced granularity, potentially complicating the differentiation between distinct systems within the same subnetwork.

In hashing, a cryptographic transformation is applied to an IP address, rendering the original address unrecoverable. For instance, when using the SHA-256 cryptographic hash function, the IP address 134.58.1.128 is transformed into the following hash: ee4e97df 20cff7a0 d33f8595 e70b28f6 ca83dabf 3c48da94 931fb13c 690d9f8c. A key advantage of this method is its consistency—identical inputs, such as the same IP address, always produce the same hash. This property enables pattern recognition while ensuring the original address remains hidden. Hashing may seemingly appear entirely secure, as the original IP address cannot be directly reconstructed. However, the risk of re-identification remains a significant concern. Given the finite and relatively small size of the IPv4 address space (contrary to IPv6), it is feasible to pre-compute hashes for every possible IPv4 address. Using such a precomputed dictionary, malicious actors could reverse a hash to reveal the original IP address, thereby compromising the intended privacy protections. This vulnerability highlights the necessity of implementing additional measures, such as salting, to strengthen confidentiality. However, public threat intelligence feeds, which often provide data in plain-text IP formats, present a challenge. For a web bot detection system utilising salted hashes, these IP addresses must be hashed with the same salt to enable effective comparisons. When a new salt is introduced, either to enhance security or to comply with rotating salt policies, the entire database of hashed IP addresses must be rehashed with the updated salt. Furthermore, all existing threat intelligence feeds need to be reprocessed to generate compatible hashes, a process that is both computationally expensive and time-consuming. Periodically rotating salts is a recommended security practice to reduce the risk of brute-force attacks or breaches. However, this practice renders historical data, such as previously hashed IP addresses, unusable unless both the salt and the original data are retained, which compromises the privacy benefits of hashing.

***5.1.2 Differential Privacy***

Differential Privacy (DP) ^63, 64^ is a privacy-preserving technique that enables statistical analysis or machine learning algorithms on datasets while minimising the risk of re-identification of individual records. The underlying principle of DP is that the output of a query (or algorithm) remains virtually the same, regardless of whether an individual record is present in the dataset. This is achieved by adding noise to the output of the algorithm, which makes it challenging to retrieve information about specific individuals. This noise is typically generated based on mathematical mechanisms such as the Laplace mechanism or the Gaussian mechanism, depending on the nature of the data and the sensitivity of the query.

In the context of web bot detection, DP can safeguard user privacy by preventing the direct association of individual records, such as a unique browser fingerprint, with specific patterns of behaviour or decisions. This is crucial since browser fingerprints are often unique and therefore carry a high risk of re-identification. DP can be applied in this scenario to obscure data by adding noise to specific attributes, such as screen resolution or user agent strings, before analysing the fingerprints. This approach makes the fingerprint less precise and more challenging to trace back to an individual.

Browser fingerprints are composed of attributes such as screen resolution, operating system, user agent, and enabled plug-ins. For instance, a fingerprint like {1920x1080, Windows 11, Chrome 131.0, no-plugins} could be unique and potentially identify an individual. Anomalies, such as an unusual combination of features (e.g., a browser pretending to be Chrome 200.0 when that version does not exist), are masked. The system introduces noise to dilute or make the anomaly in frequency data not directly detectable.

However, while DP helps prevent re-identification of unique patterns, there are several drawbacks. The added noise can reduce the accuracy of bot detection, particularly if anomalies are heavily masked. Additionally, the introduction of noise requires careful tuning to strike an appropriate balance between privacy and usability. Furthermore, aggregating rare patterns may result in the oversight of subtle yet significant anomalies.

***5.1.3 Homomorphic Encryption***

Homomorphic Encryption (HE) ^65, 66^ is a cryptographic technique that allows computations to be performed directly on encrypted data without first decrypting it. The result of these operations remains encrypted and can only be interpreted by the data owner after decryption. This approach allows sensitive data to be processed while remaining fully encrypted, significantly mitigating privacy risks. HE is specifically designed to protect sensitive data during calculations, facilitating computations without exposing the raw data. It typically requires a single processor to receive the encrypted data, perform the necessary computations, and return the encrypted output.

In the context of web bot detection, HE can be used to train and deploy machine learning algorithms without exposing sensitive data, such as browser fingerprints. Suppose a machine learning model is to be trained to differentiate bot behaviour from human behaviour based on browser fingerprints. These fingerprints include sensitive information such as the user agent, screen resolution, operating system, time zone, plug-in data, and more. Privacy protection is critical, as browser fingerprints are often unique and have the potential to identify individuals.

With HE, each party encrypts its browser fingerprint using a Fully Homomorphic Encryption (FHE) scheme before sending it to the server. For example, a browser fingerprint such as {1920x1080, Windows 11, Chrome 131.0} is transformed into its encrypted counterpart, *E*({1920x1080, Windows 11, Chrome 131.0}), with *E*() being the encryption function. During model training, the server operates solely with the encrypted fingerprints, performing computations directly on the encrypted data to train the model. The model itself also remains encrypted throughout the process, represented as *E*( *Model*), ensuring that sensitive information is never exposed. When the trained model is used for inference, such as classifying a new browser fingerprint, both the input fingerprint and the classification output remain encrypted throughout the process. After the computations are completed, the web bot detection system decrypts the classification result, converting the encrypted output *E*(“ *Bot*'') into the plaintext result “Bot”.

While HE offers robust privacy protection during the processing of sensitive data, it also presents significant challenges and limitations. The primary drawback being its computational intensity. Operations on encrypted data—such as addition or multiplication—are significantly more resource-intensive than equivalent operations on plaintext. In the case of FHE, this overhead can be orders of magnitude greater, making HE less suitable for applications requiring real-time processing or handling of large datasets. The time required for encryption, computation on encrypted data, and decryption introduces latency, which may hinder performance, particularly in scenarios such as real-time web bot detection. Additionally, implementing HE demands specialised cryptographic expertise, and integrating it with existing systems can be technically challenging.

***5.1.4 Secure Multi-Party Computation***

Secure Multi-Party Computation (SMPC) ^67, 68^ is a cryptographic technique that enables multiple parties to collaboratively perform computations on their combined data without exposing their individual data to one another. The computation result is shared, but individual inputs remains private. SMPC operates by splitting data into encrypted â€œsharesâ€, which are processed separately. Only through collaboration can the parties reconstruct the final result. Similar to HE, this technique is also designed to protect sensitive data during calculations, such as training an ML model. For instance, a browser fingerprint such as {1920x1080, Windows 11, Chrome 131.0} could be split into two shares: {960x540, Windows} and {960x540, 11, Chrome 131.0}. During model training, the servers collaborate by processing their respective shares, ensuring that no individual server has access to the complete fingerprint data. The final result of the computation can only be reconstructed by combining the outputs from all servers, ensuring that no single server can independently deduce the original data.

The main drawback of SMPC is that it requires multiple parties to participate and coordinate in the computation process. Each party must contribute its “share” of data and collaborate throughout the computation, adding a layer of organisational complexity and necessitating reliable communication channels. SMPC often involves data exchanges between parties, increasing dependence on network bandwidth and latency. High communication costs can negatively impact performance, particularly in distributed or global systems. Additionally, SMPC protocols typically assume that a subset of participants is honest or semi-honest. If these assumptions are violated, the system’s security guarantees may be undermined.

### 5.2 Limitations of PETs

Although Privacy Enhancing Technologies (PETs) provide robust technical solutions for safeguarding sensitive data, they are not a panacea and cannot mitigate all risks associated with data protection and compliance. Implementing PETs requires advanced expertise, and the complexity of these solutions can introduce vulnerabilities or errors. For example, while homomorphic encryption allows computations on encrypted data, it demands substantial computing power and resources, posing significant operational challenges, particularly in real-time applications. Furthermore, the protections offered by PETs, such as pseudonymisation, are not absolute. Data that has undergone pseudonymisation may still be considered personal data under regulations like the General Data Protection Regulation (GDPR) if there is a reasonable likelihood of re-identifying individuals. This is especially pertinent in situations where auxiliary datasets or advanced inference techniques can be employed to link pseudonymised data back to individuals.

Applicable laws ensuring respect for privacy and fundamental rights, such as the GDPR and the AI Act, require compliance with clear principles and regulations into the design and deployment of web bot detection technologies (all under the GDPR, some under the EU AI Act). While the deployment of PETs can minimise risks during data processing and analysis, they do not inherently support the transparency and auditability required by regulators. For instance, encryption or pseudonymisation technologies may safeguard data in use or at rest, but they do not offer an auditable record of how, when, or by whom the data is accessed, shared, or processed. Moreover, the mere deployment does not by itself assist in achieving other legal values protected by these design regulations, such as algorithmic bias or human oversight.

Therefore, organisations employing PETs must supplement these technical measures with strong organisational practices. This includes comprehensive documentation to demonstrate compliance, well-defined and clear policies governing the use of PETs, and regular audits to ensure their proper implementation and effectiveness. In the absence of such organisational measures, companies risk failing to meet regulatory requirements, even if advanced technical solutions are in place.

Ultimately, while PETs are critical tools in contemporary data protection strategies, their effectiveness depends on being part of a broader framework that integrates technical, organisational, and legal safeguards. This holistic approach ensures not only compliance with regulatory standards but also fosters trust among stakeholders by showcasing a commitment to privacy and data security.

## 6 Conclusions

In this paper, we have provided a detailed description of how web bot detection operates and the methods by which these detection mechanisms can be circumvented by web bots. In doing so, we highlighted the significant privacy risks involved in implementing such systems. We thoroughly analysed these challenges from the perspective of key regulations such as the GDPR and the AI Act, clarifying their potential impact and compliance requirements.

We then discussed some crucial Privacy Enhancing Technologies (PETs) and explained how they can be applied in a web bot detection context. We also highlighted their limitations, such as the fact that PETs alone do not guarantee full compliance. To comply with the GDPR and the AI Act, PETs must be supported by organisational measures, including comprehensive documentation, transparent processes, and regular audits.

We have also shown that some PETs, such as encryption and data anonymisation, present an additional challenge: they complicate the detection of evasion attacks by malicious web bots. This is due to the fact that the necessary data is often transformed or encrypted before analysis, which can result in reduced detection capabilities.

There is a need to develop new methodologies for detecting web bots, even when the data is anonymised or encrypted, such as machine learning models that remain robust against perturbed data. Additionally, it is crucial to explore how attackers may exploit PET methods and create countermeasures to identify and mitigate these attacks. Hence, future research should focus on developing solutions that are not only technically advanced, but also practically applicable and compliant with regulations. This requires a multidisciplinary approach that brings together computer science, regulation, ethics and usability.

## Ethics and consent

Ethical approval and consent were not required.

## Data Availability

No data associated with this article.
