# Supplementary material for: Balancing Security and Privacy: Web Bot Detection, Privacy Challenges, and Regulatory Compliance under the GDPR and AI Act
Source: Open Res Eur. 2025 Mar 24;5:76. [Version 1] doi: 10.12688/openreseurope.19347.1 (PMC11962364; doi:10.12688/openreseurope.19347.1)
Supplement: Peer review history

# Reviewer report: Reviewer response for version 1

**DOI:** 10.21956/openreseurope.20938.r52430

**Published:** 2025-04-01

**Authors:** Glorin Sebastian

**Affiliations:** 1 IIM Sambalpur, Sambalpur, Odisha, India

- This manuscript by Martínez Llamas and colleagues presents a comprehensive and timely analysis of the intricate relationship between web bot detection, user privacy, and relevant regulatory frameworks, specifically the EU's General Data Protection Regulation (GDPR) and the recently enacted Artificial Intelligence Act (AI Act). The authors effectively navigate the dual-edged nature of web bots, acknowledging their utility alongside the security and privacy risks they pose. The paper meticulously dissects various web bot detection methodologies, categorizing them based on their primary data sources – network traffic data, fingerprints, and behavioural biometrics – providing a structured and insightful overview of the current technological landscape.
  The discussion of advanced web bot techniques and evasion strategies is particularly well-articulated. The manuscript elucidates sophisticated methods such as headless browsers, adversarial fingerprinting, network proxies and IP rotation, rate limiting and throttling circumvention, behavioural mimicry, and the leveraging of machine learning by malicious actors. This section effectively highlights the ongoing "arms race" between detection mechanisms and evasion tactics, underscoring the dynamic and challenging nature of the field.
  A significant strength of this work lies in its thorough exploration of the privacy challenges inherent in web bot detection. The authors provide a detailed analysis of how various data points collected for detection purposes can constitute personal data under the GDPR, referencing key CJEU rulings to contextualize the legal interpretations. The discussion of legal bases for processing, the nuances of processing biometric data, and the implications of automated decision-making (Article 22 GDPR) are handled with precision and a clear understanding of the regulatory landscape. Furthermore, the anticipation of the AI Act's impact on AI-powered web bot detection systems, including the categorization of risk tiers and the obligations for high-risk systems, demonstrates a forward-thinking approach.
  The final section dedicated to Privacy Enhancing Technologies (PETs) offers a valuable perspective on potential technical solutions for mitigating privacy risks. The discussion of data minimization techniques, differential privacy, homomorphic encryption, and secure multi-party computation provides a balanced view of their capabilities and inherent limitations in the context of web bot detection. The authors astutely emphasize that PETs alone are insufficient for ensuring regulatory compliance and must be complemented by robust organizational measures.
  The conclusions drawn are well-supported by the preceding analysis. The manuscript effectively synthesizes the technical challenges, privacy implications, and regulatory requirements, highlighting the need for innovative and ethically sound approaches to web bot detection. The call for future research to focus on developing detection methodologies that are effective even with anonymized or encrypted data, while also being practically applicable and compliant with evolving regulations, is both pertinent and well-justified. The interdisciplinary nature of the challenges is rightly emphasized.
  Overall, this manuscript presents a well-researched, comprehensive, and insightful contribution to the field. The analysis is rigorous, the arguments are clearly presented, and the discussion is firmly grounded in both the technical literature and the relevant legal frameworks. The authors demonstrate a strong understanding of the complexities at the intersection of cybersecurity, privacy, and regulation. This work should be of significant interest to researchers, practitioners, and policymakers grappling with the challenges of web bot management in a privacy-conscious digital environment.

**Suggestions for potential further development (optional):**

- While the discussion of PETs is valuable, future iterations could potentially delve deeper into the practical implementation challenges and the trade-offs between security effectiveness and privacy preservation for each technology in the specific context of web bot detection.
- Exploring specific case studies or examples of how organizations are currently navigating these complex issues could further enhance the practical relevance of the paper.
- Given the rapidly evolving nature of both bot technology and AI regulations, a brief discussion of potential future trends and emerging challenges could be a valuable addition.

Is the review written in accessible language?

Yes

Are all factual statements correct and adequately supported by citations?

Yes

Are the conclusions drawn appropriate in the context of the current research literature?

Yes

Is the topic of the review discussed comprehensively in the context of the current literature?

Yes

Reviewer Expertise:

Privacy, Security, Information Systems

I confirm that I have read this submission and believe that I have an appropriate level of expertise to confirm that it is of an acceptable scientific standard.

# Reviewer report: Reviewer response for version 1

**DOI:** 10.21956/openreseurope.20938.r52436

**Published:** 2025-03-31

**Authors:** John Giordani, Renato Zeko

**Affiliations:** 1 University of Fairfax, Virginia, USA; 2 ERM, United Nations FCU, New York, New York, USA

This article provides a comprehensive interdisciplinary analysis of web bot detection techniques and the associated privacy challenges under European regulations. The article’s interpretation of regulatory frameworks is detailed and largely accurate. It appropriately identifies key provisions of the GDPR relevant to web bot detection.

 The treatment of data protection principles (Article 5 GDPR) is also evident throughout the paper. The authors implicitly address data minimization and purpose limitation by later proposing PETs that minimize data collection. They also cover transparency and fairness: notably, the manuscript considers GDPR’s provisions on automated decision-making (Article 22). With respect to the AI Act, the authors demonstrate up-to-date knowledge of this new regulation.

 One aspect of regulatory compliance that the article mentions only briefly is the ePrivacy Directive (2002/58/EC). The authors correctly note that if any bot detection technique stores or accesses data on the user’s device (for example, setting a cookie or using a browser fingerprinting script), Article 5(3) of the ePrivacy Directive requires user consent. The manuscript could be improved by elaborating slightly more on how solutions like device fingerprinting navigate ePrivacy – e.g., many sites rely on the “strictly necessary” exemption for security cookies or trackers. Clarifying this would strengthen the compliance discussion, though it may be somewhat outside the central GDPR/AI Act focus.

 The technical content of the paper is rigorous and up-to-date, offering a detailed taxonomy of web bot detection techniques and a clear-eyed look at attacker capabilities. The authors classify detection methods into three categories based on data sources - network traffic analysis, device/browser fingerprinting, and behavioral biometrics - which is a logical and comprehensive way to organize the domain. Each category is explained with sufficient background.

 Because this article is largely a literature-driven analysis and conceptual review, it does not follow a traditional experimental methodology. There is no specific “Methods” section detailing data collection or statistical analysis (indeed, none was performed). Instead, the authors undertook a broad review of technical and legal sources to construct their taxonomy and arguments. This approach is appropriate for the aims of the paper.

 An aspect of the study that cannot be fully assessed is the efficacy of the proposed balance in a real-world deployment. Since no actual system was built or tested here, the paper’s contributions are analytical. This is absolutely fine for a scholarly review, but it means we take on trust that the authors have covered the main points from all relevant angles. Given the extensive referencing and logical flow, I do trust the content. Yet, for scientific completeness, the authors might consider if there are any notable viewpoints or sources that were left out.

**Accessible language:** The review is written in an academic but generally accessible language. Given the inherently technical and legal subject matter, the authors do a commendable job explaining concepts in a way that an informed reader from either domain (computer science or privacy law) can follow. Despite this, there are spots where the language could be made more broadly accessible. The legal sections, in particular, while accurate, tend to cite regulations and case law in detail. A reader not already familiar with the GDPR or AI Act might find paragraphs dense. Phrases like “legal effects or similarly significant effects” or detailed case references could confuse non-lawyers.

Is the review written in accessible language?

Partly

Are all factual statements correct and adequately supported by citations?

Yes

Are the conclusions drawn appropriate in the context of the current research literature?

Yes

Is the topic of the review discussed comprehensively in the context of the current literature?

Yes

Reviewer Expertise:

I am a professor of computer science and a published researcher in Artificial Intelligence, with authored works in the field of machine learning. My expertise includes technology risk management, cybersecurity governance, regulatory compliance (including GDPR and the AI Act), enterprise risk management, and the integration of emerging technologies within legal and operational frameworks. I bring both academic and applied experience to the review of interdisciplinary work involving AI, data protection, and technical systems.

We confirm that we have read this submission and believe that we have an appropriate level of expertise to confirm that it is of an acceptable scientific standard.

## References

[1] : Article 5 Principles relating to processing of personal data.2020; 10.1093/oso/9780198826491.003.0034 309-320 10.1093/oso/9780198826491.003.0034

[2] : Automated Decision-Making under Article 22 GDPR.2020; 10.1017/9781108347846.005 136-156 10.1017/9781108347846.005

[3] : The Problems of Implementing the ePrivacy Directive and Their Solutions in ePrivacy Regulation. *Teisė* .2019;113: 10.15388/Teise.2019.113.8 139-154 10.15388/Teise.2019.113.8

[4] : Artificial Intelligence Act (AI Act) and the GDPR.2019; 10.1007/978-3-642-27739-9_1820-1 1-6 10.1007/978-3-642-27739-9_1820-1
